# 24-Epibrassinolide Reduces Drought-Induced Oxidative Stress by Modulating the Antioxidant System and Respiration in Wheat Seedlings

**DOI:** 10.3390/plants13020148

**Published:** 2024-01-05

**Authors:** Azamat Avalbaev, Vadim Fedyaev, Alsu Lubyanova, Ruslan Yuldashev, Chulpan Allagulova

**Affiliations:** 1 Institute of Biochemistry and Genetics—Subdivision of the Ufa Federal Research Centre of the Russian Academy of Sciences, 71 Pr. Oktyabrya, Ufa 450054, Russia; lubyanova555@mail.ru (A.L.); yuldashevra@gmail.com (R.Y.); allagulova-chulpan@rambler.ru (C.A.); 2 Institute of Nature and Human, Ufa University of Sciences and Technology, 32 Zaki Validi, Ufa 450076, Russia; vadim.fedyaev@gmail.com

**Keywords:** brassinosteroids, drought, reactive oxygen species, antioxidant enzymes, alternative respiration, *Triticum aestivum* L.

## Abstract

Brassinosteroids (BRs) represent a group of plant signaling molecules with a steroidal skeleton that play an essential role in plant adaptation to different environmental stresses, including drought. In this work, the effect of pretreatment with 0.4 µM 24-epibrassinolide (EBR) on the oxidant/antioxidant system in 4-day-old wheat seedlings (*Triticum aestivum* L.) was studied under moderate drought stress simulated by 12% polyethylene glycol 6000 (PEG). It was revealed that EBR-pretreatment had a protective effect on wheat plants as evidenced by the maintenance of their growth rate, as well as the reduction in lipid peroxidation and electrolyte leakage from plant tissues under drought conditions. This effect was likely due to the ability of EBR to reduce the stress-induced accumulation of reactive oxygen species (ROS) and modulate the activity of antioxidant enzymes. Meanwhile, EBR pretreatment enhanced proline accumulation and increased the barrier properties of the cell walls in seedlings by accelerating the lignin deposition. Moreover, the ability of EBR to prevent a drought-caused increase in the intensity of the total dark respiration and the capacity of alternative respiration contributes significantly to the antistress action of this hormone.

## 1. Introduction

Terrestrial plants, being immobile organisms, constantly experience challenges by different adverse conditions, in particular, water scarcity [1,2]. Throughout their ontogeny, plants require large amounts of water for normal functioning; therefore, a water shortage has a negative impact on multiple aspects of the whole plant metabolism [3,4]. Drought stress impairs seed germination and seedling growth and retards subsequent plant development, which ultimately results in growth reduction and a declined yield. Drought adversely affects numerous physiological and biochemical processes in plants, including cell elongation and division, water and nutrition uptake, osmotic adjustment, transpiration, photosynthesis, and respiration [5,6]. Plants have to adapt to changing environments and can develop a wide range of protective responses and adaptive mechanisms which allows for the diminishing of the detrimental effects of drought stress [2,4,7].

Plant hormones are essential signaling molecules that have been known to play a major role in the regulation of the metabolism of plant organisms and their adaptation to changing environmental conditions. Substantial progress has been achieved in understanding the role of brassinosteroids (BRs), a class of steroidal phytohormones, in the regulation of the diverse developmental and physiological processes in plants [8,9,10,11,12]. BRs are considered widespread across the plant kingdom and include more than 70 compounds found in nearly all parts and organs in free forms or conjugated with carbohydrates and fatty acids [13,14,15]. This group of plant hormones is involved in the regulation of various developmental and physiological processes, including cell division and elongation, seed development and germination, vascular system differentiation, the development of root system and reproductive organs, photomorphogenesis, flowering, and senescence [16,17,18]. Although BRs have been known for their ability to promote plant growth in extremely low concentrations [9,14,19], a lot of information has accumulated on the participation of BRs in the protective responses of plants to a wide range of adverse environmental factors [10,20,21]. Moreover, multiple studies have reported that the exogenous application of bioactive BRs improves plant tolerance to various abiotic stresses, including drought [22,23]. It has been demonstrated that the application of brassinosteroids led to the recovery of growth and yield parameters, improved photosynthetic efficiency, maintenance of redox balance, and the stability of membranes in wheat plants subjected to drought [24,25,26]. Thus, the exogenous application of brassinolide and 24-epibrassinolide enhanced the different growth (maximum plant height, spike length) and yield (grain yield, biological yield, harvest index) parameters of wheat under drought stress conditions [25,26]. Moreover, the treatment of wheat with BRs improved various physiological (relative water content, stomatal conductance, chlorophyll content) and biochemical attributes (proline, soluble carbohydrates, macroelements contents) [24,25,26]. It was shown that a BR-activated TaBZR2 transcription factor promoted the drought tolerance of wheat plants [27]. Thus, the drought-treated *TaBZR2*-overexpressing plants accumulated lower levels of reactive oxygen species (ROS) than the wild type plants. In contrast, *TaBZR2*-silenced plants exhibited drought-sensitive phenotypes. Moreover, it was found that the TaBZR2 transcription factor improved wheat drought tolerance by directly activating the expression of the glutathione S-transferase-1 (*TaGST1*) gene which encodes a ROS-scavenging enzyme [27].

The characteristic response of plants to environmental stresses is the shift in the balance of oxidants and antioxidants in favor of the increased production of oxidants [28,29,30]. Oxidants, the so-called reactive oxygen species, are normally generated during cell metabolism but are overproduced in excessive amounts under adverse conditions [31]. ROS are known to exert a dual action on plant metabolism which can be beneficial or harmful depending on the ROS concentration [32,33,34]. Thus, low levels of ROS exert beneficial effects on plant cells modulating redox state and signaling cascades. Environmental stresses cause the excessive production of ROS such as the superoxide anion (O_2_^•−^), hydroxide anion (OH^−^), and hydrogen peroxide (H_2_O_2_), which at high levels become supertoxic and actively capture electrons from biomacromolecules causing their destruction and the disturbance of plant metabolism which leads to cell death and plant growth inhibition or even its cessation [28,33,35,36,37]. ROS neutralization in plants is carried out by the antioxidant protection system, including antioxidant enzymes: superoxide dismutase, catalase, peroxidase and non-enzymatic components: glutathione, ascorbic acid, tocopherols [28,30,31,33,38].

At the same time, numerous studies have demonstrated the beneficial effects of BRs on the basic energy transformation processes in plants [39]. It has long been known that BRs exert a stimulating effect on photosynthesis, including an increase in the number and size of grana and thylakoids, the activity and quantity of Rubisco, and the quantum yield of PSII [40,41,42]. At the same time, the formation of the weight of plants depends not only on photosynthesis, but also on the intensity of the respiratory exchange. Despite a large number of publications on the positive effects of BRs on photosynthesis, there are, however, only a few studies that have reported the influence of BRs on plant respiration [43,44]. Respiration has a special role in plant adaptation to adverse conditions as the major source of energy for plant metabolic processes [45,46,47,48]. In addition to the cytochrome respiratory pathway common to all eukaryotes, plants contain the cyanide-insensitive alternative respiratory pathway catalyzed by the alternative oxidase (AOX) [49,50,51]. Several studies have reported the involvement of AOX in the neutralization of an excessive amount of ROS, as well as the critical role of alternative respiratory pathway in the adaptation of plants to different stresses [52,53]. Thus, cyanide-insensitive respiration was shown to be involved in plant protection against thermogenesis in the flowering of the sacred lotus [54] and Araceae plants [55]. Alternative oxidase is an enzyme that also has a crucial role in neutralization of the excessive amounts of ROS and the maintenance of redox homeostasis under different stresses [56]. Thus, overexpression of the alternative oxidase *AOX1a* gene in Arabidopsis plants reduced ROS production in response to aluminum treatment. Interestingly, the reduced aluminum toxicity in *AOX1a*-overexpressing plants is associated with the enhanced expression of several protective genes. Meanwhile, several studies have shown that phytohormones can regulate oxidative metabolism through the cyanide-resistant respiratory pathway [57]. Interestingly, several studies have reported the relationship between the BR signaling and AOX function under stress conditions. Thus, it has been demonstrated that plant hormones, including brassinosteroids, may enhance the AOX function under adverse environments [43,58].

In our previous study, it was found that 24-epibrassinolide (EBR) applied in a concentration of 0.4 µM had a strong promoting effect on wheat seedling growth [59]. Moreover, pretreatment with 0.4 µM EBR alleviated the injurious action of abiotic stress on wheat metabolism [60]. Drought-induced oxidative stress causes serious damage to different cellular components [37,61], and EBR’s ability to activate antioxidant defense mechanisms can contribute significantly to EBR-induced protection against water stress in wheat plants. In the present research, we have analyzed the effects of EBR on ROS generation and the activities of antioxidant enzymes, as well as on respiratory metabolism in wheat seedlings under drought stress. The obtained data suggested that treatment with EBR could improve the tolerance of wheat seedlings to drought stress and mitigate stress-induced oxidative injury. In addition, we have revealed that EBR is involved in the regulation of individual pathways of respiration (glycolysis, cytochrome oxidase and alternative pathways) that might contribute to EBR-induced drought tolerance.

## 2. Results

### 2.1. Effect of EBR Application on the Growth of Wheat Seedlings under Drought Stress

**Figure 1:**
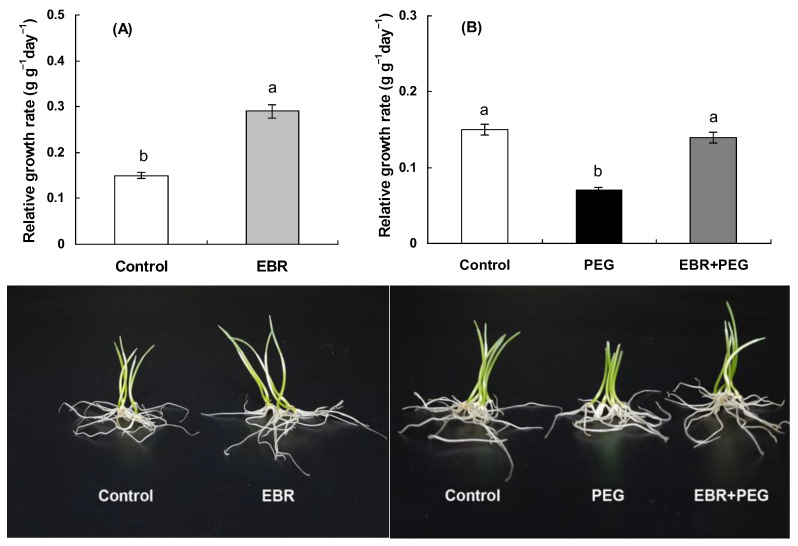
The effect of EBR on the relative growth rate and visual appearance of wheat seedlings under normal (**A**) and drought conditions (**B**). Three-day-old seedlings were placed for a day in 0.4 µM EBR solution and then were transferred to 12% PEG solution for a day. The data shown are the mean values and their standard errors (*n* = 3). Different letters indicate that means are significantly different at *p* < 0.05 (ANOVA, LSD test).

It has long been known that brassinosteroids promote plant growth and improve plant stress tolerance to different abiotic stresses, including drought [60]. The data presented in Figure 1A demonstrate that the application of 0.4 µM 24-epibrassinolide stimulated markedly the growth of wheat seedlings. Plants grown on medium with EBR were distinguished by a higher relative growth rate (RGR) compared to control plants (Figure 1A). The exposure of seedlings to polyethylene glycol 6000 (PEG) resulted in a significant reduction in their growth (Figure 1B). EBR pretreatment considerably reduced the injurious effect of drought stress on wheat plants, as was evident from the effect of the hormone on the RGR parameter (Figure 1). Thus, a low concentration of EBR (0.4 µM) was found to exert both a promoting effect and a protective action on wheat seedlings exposed to drought stress.

### 2.2. Effect of EBR on O_2_^•−^ and H_2_O_2_ Production and the Activity of Antioxidant Enzymes under Normal and Drought Conditions

**Figure 2:**
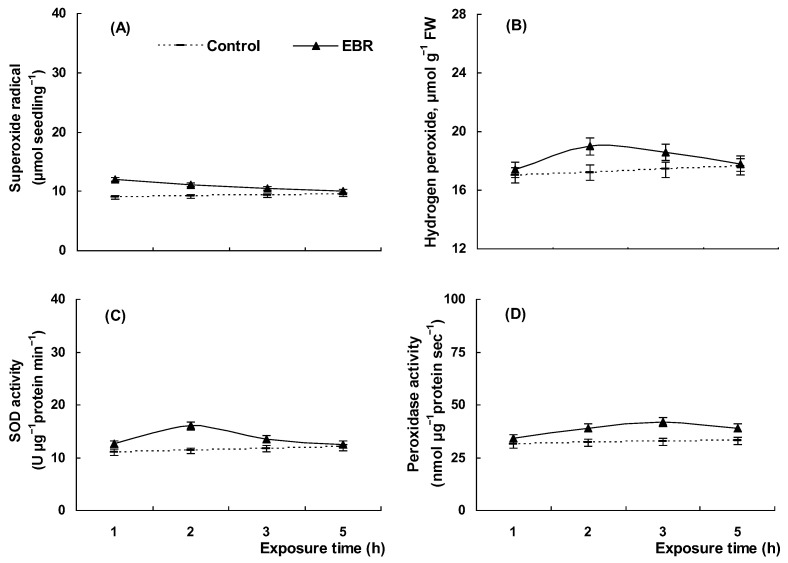
The effect of 0.4 µM EBR on O_2_^•−^ (**A**) and H_2_O_2_ (**B**) generation, SOD (**C**) and peroxidase (**D**) activity in 4-day-old wheat seedlings under normal conditions. Three-day-old seedlings were placed for a day in 0.4 µM EBR solution and then were transferred to 12% PEG solution. The data shown are the mean values and their standard errors (*n* = 3).

**Figure 3:**
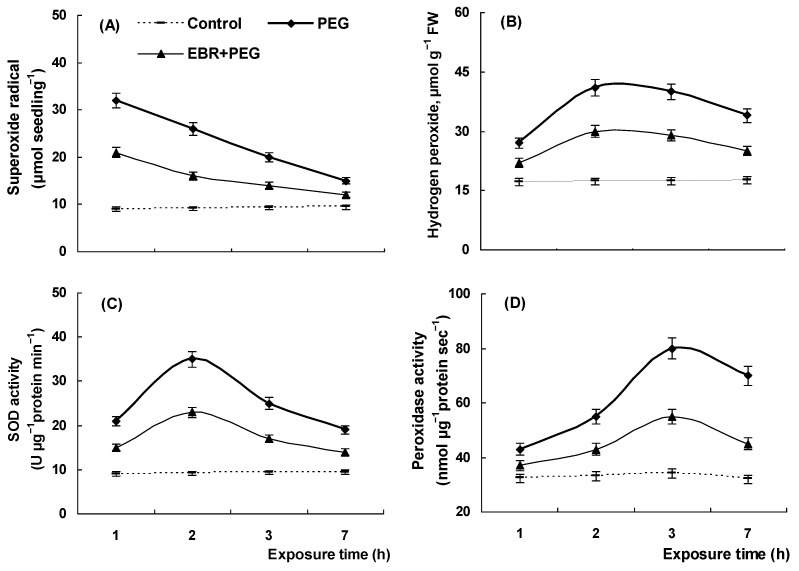
The effect of 0.4 µM EBR on O_2_^•−^ (**A**) and H_2_O_2_ (**B**) generation, SOD (**C**) and peroxidase (**D**) activity in 5-day-old wheat seedlings under drought conditions. Three-day-old seedlings were placed for a day in 0.4 µM EBR solution and then were transferred to 12% PEG solution. The data shown are the mean values and their standard errors (*n* = 3).

The incubation of seedlings in the medium containing 0.4 μM EBR resulted in a slight reversible acceleration of O_2_^•−^ production (Figure 2A) followed by an insignificant increase in H_2_O_2_ content (Figure 2B). Simultaneously with the ROS generation, there was light activation of antioxidant enzymes—superoxide dismutase (SOD) which catalyzed O_2_^•−^ conversion into H_2_O_2_ and peroxidase which is involved in the scavenging of H_2_O_2_ (Figure 2C,D). The exposure of seedlings to PEG solution caused a rapid almost 4-fold enhancement in the O_2_^•−^ content (Figure 3A). Then, a significant rise in H_2_O_2_ levels was observed during PEG application, and the hydrogen peroxide concentration in drought-stressed plants was higher than that of the control ones until the end of the stress treatment (Figure 3B). As shown in Figure 3, elevated ROS production was accompanied by a significant increase in SOD and then in peroxidase activity in seedlings under drought stress. The concentrations of O_2_^•−^ and H_2_O_2_ in seedlings pretreated with EBR and incubated in 12% PEG solution were substantially less than in hormone-untreated drought-stressed seedlings (Figure 3A,B). In addition, pretreatment with EBR significantly reduced the stress-induced rise in SOD and peroxidase activities (Figure 3C,D). Although 12% PEG also caused an increase in the activity of antioxidant enzymes in EBR-pretreated seedlings, the magnitude of this effect was much smaller than that of the hormone-untreated seedlings (Figure 3C,D).

### 2.3. Effect of EBR on Proline under Normal and Drought Conditions

**Figure 4:**
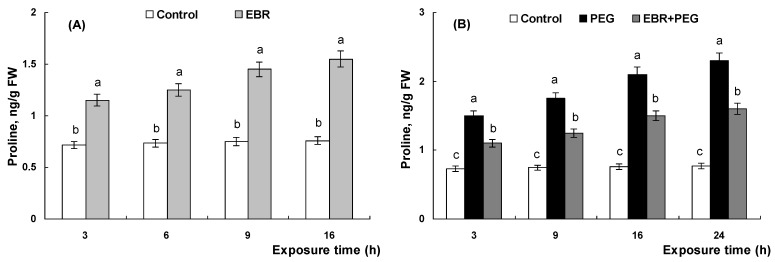
The proline content in wheat seedlings pretreated and untreated with EBR under normal (**A**) and drought (**B**) conditions. Three-day-old seedlings were placed for a day in 0.4 µM EBR solution and then were transferred to 12% PEG solution for a day. The data shown are the mean values and their standard errors (*n* = 3). Different letters indicate that the means are significantly different at *p* < 0.05 (ANOVA, LSD test).

Exogenous application of EBR induced the changes in the content of proline under both normal and drought conditions (Figure 4). The proline concentration was increased in wheat seedlings in response to the hormone (Figure 4A). Drought caused a significant enhancement in the proline level, which increased almost 2-fold by 3 h of stress treatment (Figure 4B). Further accumulation of this osmoprotectant was observed by the end of the experiment. Seedling pretreatment with EBR led to a considerable decline in the proline level under drought conditions as compared to the seedlings exposed to polyethylene glycol alone (Figure 4B).

### 2.4. Effect of EBR on the Respiration Parameters of Wheat Seedlings under Normal and Drought Conditions

**Figure 5:**
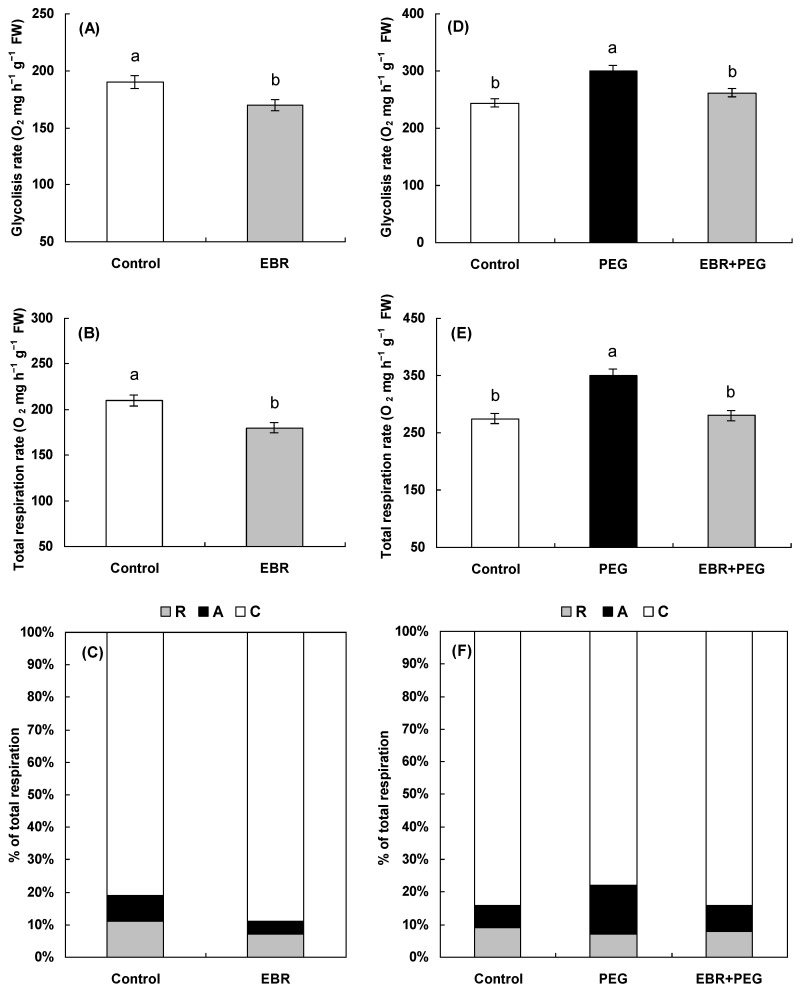
The effect of 0.4 µM EBR on the glycolysis rate, total respiration rate, and capacity of individual pathways of respiration—cytochrome oxidase (C), alternative (cyanide-resistant) (A), and residual respiration (R)—in wheat seedlings under normal (**A**–**C**) and drought (**D**–**F**) conditions. Three-day-old seedlings were placed for a day in 0.4 µM EBR solution and then were transferred to 12% PEG solution for a day. The data shown are the mean values and their standard errors (*n* = 3). Different letters indicate that the means are significantly different at *p* < 0.05 (ANOVA, LSD test).

The incubation of wheat plants in EBR solution for 24 h caused a decrease in the rate of glycolysis (Figure 5A). The EBR treatment also reduced the rate of total dark respiration (Figure 5B). It should be noted that the decline in total respiration was accompanied by a decrease in the share of energetically less effective alternative respiration (by 50%) and of residual respiration (by 39%) (Figure 5C).

As shown in Figure 5D–F, drought considerably increased the rates of glycolysis and total respiration whereas the share of alternative respiration was doubled in comparison with the control. Pretreatment with EBR for 24 h prevented the stress-induced increase in glycolysis and reduced the rate of total respiration (Figure 5D,E). Under drought conditions, the proportion of the cyanide-resistant pathway remained at a low level in EBR-pretreated plants compared to hormone-untreated (Figure 5F).

### 2.5. Lignin Deposition in EBR-Pretreated and EBR-Untreated Wheat Seedlings under Normal and Drought Conditions

It is known that the enhanced synthesis and deposition of lignin in the cell walls considerably contributes to the improvement of plant barrier properties under stress conditions [62]. As shown in Table 1, the treatment of seedlings with EBR for 24 h stimulated lignification of the root cell walls. In the stele of PEG-treated wheat roots, the cell walls were found to be more lignified than those of control seedlings. Meanwhile, EBR-pretreatment caused further enhancement of the lignification in seedling roots induced by drought stress (Table 1).

### 2.6. Influence of Drought Stress on Membrane Lipid Peroxidation and Electrolyte Leakage of Wheat Seedlings Untreated and Pretreated with EBR

Table 2 shows that the treatment of wheat seedlings with 0.4 μM EBR led to insignificant changes in the concentration of malondialdehyde (MDA), one of the final products of lipid peroxidation, and electrolyte leakage from the tissues. Drought exerted a damaging effect on plant membranes as evidenced by a strong increase in the indices for lipid peroxidation and the membrane permeability of seedlings (Table 2). Pretreatment with EBR for 24 h had a protective effect on wheat seedlings under water stress as can be seen from the reduced MDA content and the leakage of electrolytes (Table 2).

## 3. Discussion

Various environmental stresses cause an imbalance in the oxidant/antioxidant homeostasis in plant cells which is associated with the overproduction of reactive oxygen species (ROS) causing the growth inhibition, the damage to cell membrane structures, and an increase in membrane permeability [28,38]. In this work, it was demonstrated that the incubation of wheat seedlings in the 12% PEG medium resulted in a significant increase in ROS generation (Figure 3A,B) followed by significant increase in SOD and peroxidase activities (Figure 3C,D). The drought-induced oxidative stress exerted an injurious effect on wheat seedlings as indicated by the enhanced MDA and electrolyte leakage values (Table 2) and a substantial decrease in the relative growth rate observed in stressed plants (Figure 1B).

Numerous experiments with different plant species showed the efficiency of BR application either by seed priming or treatment during vegetative phases with the aim of increasing plant productivity and tolerance under the action of adverse environmental conditions, including dehydration [24,63,64,65]. A lot of studies have reported the ability of brassinosteroids to modulate the activity of antioxidant enzymes under adverse environments thus maintaining the oxidant/antioxidant homeostasis in plants [10,24,65]. We demonstrated that EBR application mitigated the drought-induced oxidative stress in wheat seedlings (Figure 3). Indeed, the concentrations of O_2_^•−^ and H_2_O_2_ and activities of SOD and peroxidase in seedlings pretreated with EBR and incubated in 12% PEG solution were noticeably less than in hormone-untreated stressed seedlings (Figure 3A,B). Moreover, under drought conditions, EBR-pretreated seedlings had reduced levels of membrane lipid peroxidation and electrolyte leakage (Table 2) and were distinguished by the recovery of growth (Figure 1B).

It is noteworthy that the application of EBR under normal conditions resulted in a minor transient ROS accumulation, as well as an insignificant increase in the activities of antioxidant enzymes (Figure 2). However, EBR-induced low levels of ROS did not cause oxidative damage to seedlings, which is illustrated by the absence of noticeable changes in the concentration of MDA and electrolyte leakage from tissues (Table 2) and by the pronounced growth-promoting effect of EBR on plants (Figure 1A). At the same time, a low level of ROS generation may play an important role in the EBR-induced preadaptation of wheat seedlings to subsequent stress because, at low concentrations, ROS are known to act as secondary messengers triggering an acclimation response [38] that would ameliorate the effects of drought on metabolism and eliminate the overproduced ROS. Thus, the EBR-induced acclimation response, which may include the activation of antioxidant enzymes, enhancement of lignin deposition, and accumulation of the defense compound proline, would promote an effective neutralization of the forthcoming drought-induced oxidative stress. Indeed, present research has demonstrated that EBR-pretreated drought-stressed seedlings experience less oxidative damage than hormone-untreated plants as evidenced by lower levels of ROS, MDA accumulation, and leakage of electrolytes (Figure 3A,B, Table 2).

Plant species respond to environmental stressors, including drought, by accumulating osmoprotectants such as proline which enable plants to overcome adverse conditions [66]. Many studies have shown that the proline content is modulated by phytohormones, including brassinosteroids [24,67]. In the present study, EBR treatment was shown to increase proline content in wheat seedlings under normal conditions (Figure 4) suggesting that elevated levels of proline may contribute considerably to EBR-induced tolerance of wheat plants against oxidative stress. Indeed, a strong correlation between elevated levels of proline and enhanced antioxidant responses has been reported under different stresses [68,69]. Proline is also involved in plant protection from oxidative stress by detoxification of harmful ROS like superoxide ions, hydrogen peroxide, and other free radicals [70,71]. Moreover, proline participates in cellular osmotic adjustment and stabilization of macromolecules and membrane structures of plants under the adverse action of a wide range of abiotic stresses. A lot of studies have shown that the exogenous application of proline can considerably reduce ROS levels and the membrane damage induced by abiotic stresses [70]. Thus, foliar application of proline significantly improved the growth and yield characteristics of cowpea plants under drought stress by reducing the MDA content and improving biochemical attributes such as SOD, peroxidase, and catalase activities [72]. Moreover, transgenic plants with an overexpression of proline biosynthesis genes are characterized by reduced ROS levels and lipid peroxidation [73]. It should be noted, however, that EBR-pretreated stressed seedlings had a reduced level of drought-induced proline accumulation as compared to the proline level in stressed seedlings untreated with the hormone (Figure 4B). This finding may be explained by the assumption that EBR-pretreated seedlings have experienced lower levels of drought damage as confirmed by their improved relative growth rate compared to that of untreated stressed seedlings (Figure 1B). Earlier, it was demonstrated that EBR pretreatment had a protective effect on rice seedlings under salinity stress. The stress-induced proline accumulation in EBR-pretreated seedlings was also below that of in hormone-untreated salt-stressed seedlings [74].

Lignin is a highly branched polymer composed of phenylpropanoid compounds deposited in plant cell walls. Lignin has an important role in plant adaptation to different stress factors, including drought stress. It enhances the rigidity and hydrophobic properties of plant cell walls, thus markedly strengthening their barrier properties. Moreover, lignin accumulation retains the normal functioning of tracheary elements in the xylem, thus maintaining water transport under drought conditions [75,76,77]. In this study, EBR-treated wheat seedlings exhibited an increased lignin deposition in their cell walls (Table 1). At the same time, drought-stressed plants also show enhanced lignin content. Importantly, under drought stress, additional lignification was revealed within the cell walls of EBR-pretreated seedlings in comparison with that of hormone-untreated seedlings (Table 1). The ability of EBR to enhance lignin deposition may help to reduce the harmful effects of drought stress on plant cells. This is evidenced by the lowered indices of MDA and electrolyte leakage in EBR-pretreated wheat seedlings compared to the hormone-untreated seedlings under drought conditions (Table 2). These data indicate that the additional accumulation of lignin may confer the protection of cells from the adverse effects of drought stress.

It has long been known that respiration is a multicomponent process including several pathways differing in energetic efficiency [45,78]. The changes in the growth rate and oxidant/antioxidant balance are considered to be energetically costly processes directly related to respiration. It should be noted that the observed effect of EBR on the oxidant/antioxidant homeostasis of wheat seedlings suggested the ability of this hormone to actively influence the energy balance of seedlings which underlies their growth-stimulating action in normal conditions and a protective effect under drought stress. Using inhibitory analysis, we investigated the capacity of individual pathways of dark respiration in EBR-treated wheat seedlings: cytochrome oxidase pathway, alternative (cyanide-resistant) pathway, and residual respiration, which in turn allowed us to indirectly estimate the energy efficiency of respiration.

It should be noted that the seedlings treated with EBR were characterized by a decrease in the intensity of total respiration and glycolysis (Figure 5A,B). It may be suggested that reduced respiration promotes the maintenance of energy equivalents that subsequently may provide additional energy under adverse conditions. Moreover, a decrease in total respiration was accompanied by a 2-fold decrease in the share of an energetically less effective alternative cyanide-resistant respiratory pathway (Figure 5C) which was accompanied by the growth-promoting action of the hormone (Figure 1A). Thus, the growth stimulation of wheat seedlings has occurred on the background of suppression of an energetically less effective alternative respiration pathway and residual respiration that is not associated with the formation of energy equivalents.

Drought evoked a noticeable increase in the total respiration and the intensity of glycolysis (Figure 5D,E) accompanied by the inhibition of the growth rate of seedlings (Figure 1B). Respiration is believed to have an essential role in plant adaptation to stress conditions as the source of energy for plant metabolism [45]. By inhibiting metabolic activity and plant growth, adverse factors can enhance respiration for reparation processes providing resources for the synthesis and accumulation of protective compounds [78,79]. Moreover, stress-induced enhanced respiration is associated with additional respiratory costs for stress adaptation and dissipation processes under stress [78,79]. Indeed, drought exposure is thought to lead to elevated energy requirements and increased respiration [80]. It should be noted that stressed wheat plants were distinguished by a two-fold increase in the capacity of the alternative respiratory pathway relative to the control (Figure 5F), which, apparently, is important for plants under oxidative stress due to the detection of antioxidant activity in cyanide-resistant oxidase [81]. Moreover, alternative oxidase has been considered to have a role in improving plant tolerance to multiple environmental stresses [52,53]. Indeed, it was shown that the alternative respiration rate was significantly increased in stressful conditions, for instance, under salt [82], cold [83], aluminum [84], and drought [85] stresses. Alternative oxidase was shown to be involved in the development of cold tolerance in winter wheat seedlings [86]. Several studies have reported a role for alternative respiration in plant responses against Cd stress [87,88]. Moreover, changes in the AOX activity in *A. thaliana* plants can affect plant protective responses by modulating redox homeostasis under Cd toxicity [89].

At the same time, pretreatment with EBR prevented the stress-induced increase in total respiration, markedly reduced the rate of glycolysis, and halved the proportion of the cyanide-resistant respiratory pathway (Figure 5D–F). Based on these facts, it can be assumed that the EBR-pretreated plants were likely to experience a less negative effect under drought stress than hormone-untreated seedlings. Indeed, EBR-pretreated seedlings appeared to be more tolerant as evidenced by a decrease in drought-induced ROS production and maintenance of the oxidant/antioxidant balance and growth at a level close to the control in these seedlings. It is assumed that more tolerant plants may be able to control their respiration rate or use more efficient respiratory pathways [90,91]. For instance, high temperature led to a 30% rise in respiration rates in two species, *Dactylis glomerata* and *Poa annua*, but *Bellis perennis* plants better acclimated to hyperthermia maintained a slower respiration rate than the other two species [90]. Thus, the experiments have revealed the activation of an alternative respiration pathway in the response of plants to drought-induced oxidative stress, while pretreatment with EBR promoted a two-fold reduction in the capacity of the energetically less favorable alternative respiration pathway (Figure 5F). This might be a part of the mechanism of BR-induced drought tolerance of wheat plants. Earlier experiments with *A. thaliana* plants have revealed the participation of alternative oxidase in the BR-mediated adaptation response to drought-induced oxidative stress [43].

Thus, EBR treatment improved the drought tolerance of wheat plants by modulating the oxidant/antioxidant homeostasis and increasing respiratory efficiency by lowering the capacity of the alternative respiration pathway under drought conditions.

## 4. Materials and Methods

### 4.1. Plant Material and Experimental Design

The seeds of spring wheat (*Triticum aestivum* L.) cv. Ekada 70 were supplied by Chishminsky Breeding Station, Bashkortostan, Russia. The wheat seeds were subjected to surface sterilization with 96% ethanol and then washed thoroughly with tap water. After that, seeds were germinated for 3 days on filter paper moistened with tap water at an irradiance of 200 µmol m^−2^ s^−1^, a 16 h photoperiod, and a 22–24 °C temperature. After the removal of endosperm, one group of 3-day-old seedlings was placed into 2% sucrose solution for a day. Thereafter, 4-day-old seedlings were incubated in 2% sucrose solution supplemented with 0.4 µM EBR for different time intervals. The stock solution of hormone (2 mM EBR in 96% ethanol) was diluted to obtain the effective EBR concentration at 0.4 µM which was selected previously [92]. For the stress assay, another group of the 3-day-old seedlings was incubated in 2% sucrose solution supplemented with 0.4 µM EBR for a day. After that, EBR-pretreated 4-day-old seedlings were grown in a drought-causing medium containing 2% sucrose and 12% polyethylene glycol 6000 (PEG) for different time intervals [93]. Wheat seedlings grown in a 2% sucrose solution were used as a control.

### 4.2. Growth Rate Analysis

The growth was estimated by the increments in fresh weight of wheat plants pretreated with EBR and subjected to 12% PEG for a day. The relative growth rate (RGR, g g^−1^ day^−1^) was calculated by the following formula: RGR = (ln FW2 − ln FW1)/Δt, where FW1 = fresh weight at the beginning of the treatment, and FW2 = final fresh weight, and Δt = time of treatment. Growth assays were conducted in three biological repeats; 50 seedlings were used for each variant.

### 4.3. Determination of Superoxide Radical and Hydrogen Peroxide Production

The production of superoxide anion (O_2_^•−^) was detected by monitoring the oxidation of epinephrine (Sigma-Aldrich, St. Louis, MO, USA) to adrenochrome as previously stated in [94]. For this purpose, 1 mL of 10^−3^ M solution of epinephrine was added to both control and experimental samples (10 seedlings each) after they had been incubated for 60 min at 30 °C in a 0.025 mM solution of CaCl_2_. The reaction of epinephrine conversion to adrenochrome was halted after 15 min of seedling incubation by adding 0.05 M HCl. The absorbance of samples was detected spectrophotometrically at 490 nm.

The content of hydrogen peroxide (H_2_O_2_) was measured as described in [95]. For this purpose, seedling samples were homogenized in 50 mM sodium phosphate buffer (pH 6.2), after which homogenates were centrifuged at 10,000× *g* for 15 min. The concentration of H_2_O_2_ in the supernatant was detected spectrophotometrically at 560 nm.

### 4.4. Determination of Antioxidant Enzymes Activities

The activity of superoxide dismutase (EC: 1.15.1.1.) was measured using the nitroblue tetrazolium photoreduction method [96]. For the analysis of total SOD activity, 0.25 g of frozen sample was ground in 0.005 M Tris-HCl buffer (pH 7.4) at 20 °C to obtain a homogenate. After centrifugation of the homogenate at 10,000× *g* for 15 min at 5 °C, the supernatant was used for the assay of SOD. One unit (U) of SOD activity was defined as the amount of enzyme required for the inhibition of nitroblue tetrasolium photoreduction by 50%. The peroxidase (EC: 1.11.1.7) activity assay was carried out according to Yusupova et al. [97]. Control and experimental seedling samples (0.25 g) were ground using 0.01 M Na-phosphate buffer (pH 6.0), then the obtained enzyme extracts were centrifuged at 14,000× *g* for 10 min. The supernatant was incubated in 0.5 mg/mL o-phenylendiamine solution. After that, H_2_O_2_ was added to the mixture for 1 min and the reaction was stopped by the addition of 2 M H_2_SO_4_, and the absorbance value was measured at 492 nm. The protein concentration was assayed following the method of Bradford [98].

### 4.5. Evaluation of Lignin Deposition

The lignification of cell walls was estimated by the intensity of lignin staining with phloroglucinol-HCl [99]. Histochemical tests were made with hand-cut sections (0.5 mm thickness) prepared from the basal parts of wheat seedling roots. The Wiesner reaction was performed by soaking the sections in phloroglucinol-ethanol solution for 3 min, and adding one drop of concentrated HCl. The intensity of lignin deposition was assayed on a scale of staining intensity.

### 4.6. Estimation of Malondialdehyde and Electrolyte Leakage

The content of malondialdehyde, one of the main products of lipid peroxidation, was measured according to [21]. For this purpose, 1 g of seedling sample was ground in 10% trichloroacetic acid (TCA). After centrifugation of the extracts at 12,000× *g* for 20 min, thiobarbituric acid in 20% TCA was added to an equal volume of the supernatant, and the mixture was boiled for 30 min, followed by centrifugation at 8000× *g* for 15 min. After centrifugation, the optical density of the supernatant was recorded at 532 nm. Estimation of electrolyte leakage from the seedlings tissues is a well-known method for evaluating the membrane permeability [100]. For this purpose, 1 g of fresh seedling sample was washed by tap water, cut, and incubated in distilled water at 27 °C during 1 h. After filtration, the electrolyte leakage of the water extract was detected with the conductivity meter HI8733 (Hanna Instruments, Inc., Woonsocket, RI, USA).

### 4.7. Proline Determination

The concentration of proline was evaluated following Bates et al. [101]. Briefly, seedling samples (0.5 g) were ground in 5 mL of 3% sulphosalicylic acid and the mixture was centrifuged at 12,000× *g* for 15 min. Then, 2 mL of acid-ninhydrin and 2 mL of glacial acetic acid were added to 2 mL of supernatant. The mixture was heated in a water bath at 100 °C for 60 min and cooled at room temperature. The absorbance was detected at 520 nm. A previously obtained calibration curve was used for the evaluation of proline content.

### 4.8. Determination of Respiration Parameters

The rates of glycolysis and total dark respiration were determined manometrically in a Warburg apparatus [102]. For this purpose, whole wheat seedlings were cut into fragments of 2–3 mm in length. Average samples (300 mg of fresh material) were taken from 30 to 50 plants and placed in manometric vessels.

Inhibitory analysis was utilized to examine the functioning of the individual pathways of dark respiration: cytochrome oxidase and alternative pathways, and residual respiration. Cytochrome oxidase and alternative pathways were inhibited by 2 mM NaN_3_ (pH 4.5) and 15 mM salicylhydroxamic acid (pH 4.5), respectively. Residual respiration was estimated in the presence of both inhibitors. The concentrations of each inhibitor were selected earlier [78].

### 4.9. Statistical Analysis

All experiments were performed in triplicate and the experimental data were subjected to a one-way analysis of variance (ANOVA) using the SPSS 13.0 software (SPSS Inc., Chicago, IL, USA). The data shown in Figures and Tables are the mean values and their standard errors (*n* = 3). The significance of differences between the mean values was evaluated by the least significant difference (LSD) test at *p* < 0.05.

## 5. Conclusions

The present study has shown that the exogenous application of 24-epibrassinolide efficiently alleviated PEG-induced drought stress in wheat seedlings. Pretreatment with EBR improved the growth rate and reduced the MDA and electrolyte leakage indices under drought conditions. This beneficial effect of EBR was achieved due to the ability of the hormone to diminish the drought-induced rise in ROS levels by modulating the activity of antioxidant enzymes. Moreover, EBR pretreatment induced proline accumulation and accelerated lignin deposition, thus enhancing the barrier properties of the cell walls in seedlings. Moreover, the application of EBR was found to increase the respiratory efficiency of wheat seedlings by lowering the capacity of the alternative respiration pathway that contributes significantly to the protective action of EBR against drought stress.

## Figures and Tables

**Table 1:** Qualitative analysis of lignin staining by Wiesner (phloroglucinol-HCl) test in root sections of 5-day-old wheat seedlings pretreated and untreated with EBR and exposed to 12% PEG *.

Variant	5th Day
Control	–
EBR	+
12% PEG	+
EBR + 12% PEG	++

* Three-day-old seedlings were incubated in 0.4 µM EBR solution for a day and then were exposed to 12% PEG solution for a day; “–” indicates the absence of staining by phloroglucinol-HCl; the number of “+” displays the extent of staining intensity.

**Table 2:** The impact of drought stress on malondialdehyde content and electrolyte leakage of 5-day-old wheat seedlings pretreated and untreated with EBR *.

Variant	MDA Content, mol g^−1^ Fresh Weight	Electrolyte Leakage, µS/g Fresh Weight
Control	40.8 ± 2.0 c	20.1 ± 1.3 c
EBR	42.2 ± 1.9 c	21.9 ± 1.4 c
12% PEG	95.5 ± 4.2 a	47.8 ± 2.3 a
EBR + 12% PEG	65.1 ± 3.1 b	32.3 ± 1.7 b

* Three-day-old wheat seedlings were placed in 0.4 µM EBR solution for a day and then were subjected to 12% PEG for a day. The data shown are the mean values and their standard errors (*n* = 3). Different letters indicate that means in the same column are significantly different at *p* < 0.05 (ANOVA, LSD test).

## Data Availability

Data are contained within the article.

## References

[1] Díaz K., Espinoza L., Carvajal R., Silva-Moreno E., Olea A.F., Rubio J. (2021). Exogenous Application of Brassinosteroid 24-Norcholane 22(S)-23-Dihydroxy Type Analogs to Enhance Water Deficit Stress Tolerance in *Arabidopsis thaliana*. Int. J. Mol. Sci..

[2] Dietz K.J., Zörb C., Geilfus C.M. (2021). Drought and crop yield. Plant Biol..

[3] Osakabe Y., Osakabe K., Shinozaki K., Tran L.-S.P. (2014). Response of plants to water stress. Front. Plant Sci..

[4] Hura T., Hura K., Ostrowska A. (2023). Drought-stress induced physiological and molecular changes in plants 2.0. Int. J. Mol. Sci..

[5] Razi K., Muneer S. (2021). Drought stress-induced physiological mechanisms, signaling pathways and molecular response of chloroplasts in common vegetable crops. Crit. Rev. Biotechnol..

[6] González E.M. (2023). Drought stress tolerance in plants. Int. J. Mol. Sci..

[7] Farooq M., Wahid A., Kobayashi N., Fujita D., Basra S.M.A. (2009). Plant drought stress: Effects, mechanisms and management. Agron. Sustain. Dev..

[8] Khripach V., Zhabinskii V., de Groot A. (2000). Twenty years of brassinosteroids: Steroidal plant hormones warrant better crops for the XXI century. Ann. Bot..

[9] Sasse J.M. (2003). Physiological actions of brassinosteroids: An update. J. Plant Growth Regul..

[10] Bajguz A., Hayat S. (2009). Effects of brassinosteroids on the plant responses to environmental stresses. Plant Physiol. Biochem..

[11] Tang J., Han Z., Chai J. (2016). Q&A: What are brassinosteroids and how do they act in plants?. BMC Biol..

[12] Lanctot A. (2022). Branching out underground: Brassinosteroid signaling promotes lateral root development in rice. Plant Physiol..

[13] Bajguz A., Tretyn A. (2003). The chemical characteristic and distribution of brassinosteroids in plants. Phytochemistry.

[14] Kutschera U., Wang Z.Y. (2012). Brassinosteroid action in flowering plants: A Darwinian perspective. J. Exp. Bot..

[15] Sadura I., Janeczko A. (2018). Physiological and molecular mechanisms of brassinosteroid-induced tolerance to high and low temperature in plants. Biol. Plant.

[16] Müssig C., Altmann T. (2003). Genomic brassinosteroid effects. J. Plant Growth Regul..

[17] Filek M., Sieprawska A., Kościelniak J., Oklestkova J., Jurczyk B., Telk A., Biesaga-Kościelniak J., Janeczko A. (2019). The role of chloroplasts in the oxidative stress that is induced by zearalenone in wheat plants—The functions of 24-epibrassinolide and selenium in the protective mechanisms. Plant Physiol. Biochem..

[18] Kim E.J., Russinova E. (2020). Brassinosteroid signalling. Curr. Biol..

[19] Jager C.E., Symons G.M., Ross J.J., Reid J.B. (2008). Do brassinosteroids mediate the water stress response?. Physiol. Plant..

[20] Gruszka D. (2020). Exploring the Brassinosteroid Signaling in Monocots Reveals Novel Components of the Pathway and Implications for Plant Breeding. Int. J. Mol. Sci..

[21] Manghwar H., Hussain A., Ali Q., Liu F. (2022). Brassinosteroids (BRs) Role in Plant Development and Coping with Different Stresses. Int. J. Mol. Sci..

[22] Ahmad Lone W., Majeed N., Yaqoob U., John R. (2022). Exogenous brassinosteroid and jasmonic acid improve drought tolerance in *Brassica rapa* L. genotypes by modulating osmolytes, antioxidants and photosynthetic system. Plant Cell Rep..

[23] Yao T., Xie R., Zhou C., Wu X., Li D. (2023). Roles of Brossinosteroids Signaling in Biotic and Abiotic Stresses. J. Agric. Food Chem..

[24] Tanveer M., Shahzad B., Sharma A., Khan E.A. (2019). 24-Epibrassinolide application in plants: An implication for improving drought stress tolerance in plants. Plant Physiol. Biochem..

[25] Dehghan M., Balouchi H., Yadavi A., Zare E. (2020). Improve wheat (*Triticum aestivum*) performance by brassinolide application under different irrigation regimes. S. Afr. J. Bot..

[26] Raza M.A.S., Ibrahim M.A., Ditta A., Iqbal R., Aslam M.U., Muhammad F., Ali S., Çiğ F., Ali B., Muhammad Ikram R. (2023). Exploring the recuperative potential of brassinosteroids and nano-biochar on growth, physiology, and yield of wheat under drought stress. Sci. Rep..

[27] Cui X.-Y., Gao Y., Guo J., Yu T.-F., Zheng W.-J., Liu Y.-W., Chen J., Xu Z.-S., Ma Y.-Z. (2019). BES/BZR transcription factor TaBZR2 positively regulates drought responses by activation of TaGST1. Plant Physiol..

[28] Demidchik V. (2015). Mechanisms of oxidative stress in plants: From classical chemistry to cell biology. Environ. Exp. Bot..

[29] Wakeel A., Xu M., Gan Y. (2020). Chromium-Induced Reactive Oxygen Species Accumulation by Altering the Enzymatic Antioxidant System and Associated Cytotoxic, Genotoxic, Ultrastructural, and Photosynthetic Changes in Plants. Int. J. Mol. Sci..

[30] Mansoor S., Ali Wani O., Lone J.K., Manhas S., Kour N., Alam P., Ahmad A., Ahmad P. (2022). Reactive Oxygen Species in Plants: From Source to Sink. Antioxidants.

[31] Farooq M.A., Niazi A.K., Akhtar J., Saifullah, Farooq M., Souri Z., Karimi N., Rengel Z. (2019). Acquiring control: The evolution of ROS-induced oxidative stress and redox signaling pathways in plant stress responses. Plant Physiol. Biochem..

[32] Singh R., Singh S., Parihar P., Mishra R.K., Tripathi D.K., Singh V.P., Chauhan D.K., Prasad S.M. (2016). Reactive oxygen species (ROS): Beneficial companions of plants’ developmental processes. Front. Plant Sci..

[33] Huang H., Ullah F., Zhou D.X., Yi M., Zhao Y. (2019). Mechanisms of ROS regulation of plant development and stress responses. Front. Plant Sci..

[34] Li W., Niu Y., Zheng Y., Wang Z. (2022). Advances in the understanding of reactive oxygen species-dependent regulation on seed dormancy, germination, and deterioration in crops. Front. Plant Sci..

[35] Hossain M.A., Bhattacharjee S., Armin S.M., Qian P., Xin W., Li H.Y., Burritt D.J., Fujita M., Tran L.S. (2015). Hydrogen peroxide priming modulates abiotic oxidative stress tolerance: Insights from ROS detoxification and scavenging. Front. Plant Sci..

[36] Mittler R. (2017). ROS are good. Trends Plant Sci..

[37] Hasanuzzaman M., Bhuyan M.H.M.B., Zulfiqar F., Raza A., Mohsin S.M., Mahmud J.A., Fujita M., Fotopoulos V. (2020). Reactive Oxygen Species and Antioxidant Defense in Plants under Abiotic Stress: Revisiting the Crucial Role of a Universal Defense Regulator. Antioxidants.

[38] Choudhury F.K., Rivero R.M., Blumwald E., Mittler R. (2017). Reactive oxygen species, abiotic stress and stress combination. Plant J..

[39] Liu Z., Li L., Luo Z., Zeng F., Jiang L., Tang K. (2016). Effect of brassinolide on energy status and proline metabolism in postharvest bamboo shoot during chilling stress. Postharvest Biol. Technol..

[40] Yu J.Q., Huang L.F., Hu W.H., Zhou Y.H., Mao W.H., Ye S.F., Nogueas S. (2004). A role for brassinosteroids in the regulation of photosynthesis in *Cucumis sativus*. J. Exp. Bot..

[41] Xia X.-J., Huang L.-F., Zhou Y.-H., Mao W.-H., Shi K., Wu J.-X., Asami T., Chen Z., Yu J.-Q. (2009). Brassinosteroids promote photosynthesis and growth by enhancing activation of Rubisco and expression of photosynthetic genes in *Cucumis sativus*. Planta.

[42] Shu S., Tang Y., Yuan Y., Sun J., Zhong M., Guo S. (2016). The role of 24-epibrassinolide in the regulation of photosynthetic characteristics and nitrogen metabolism of tomato seedlings under a combined low temperature and weak light stress. Plant Physiol. Biochem..

[43] Derevyanchuk M.V., Grabelnyh O.I., Litvinovskaya R.P., Voinikov V.K., Sauchuk A.L., Khripach V.A., Kravets V.S. (2014). Influence of brassinosteroids on plant cell alternative respiration pathway and antioxidant systems activity under abiotic stress conditions. Biopolym. Cell.

[44] Zhu T., Deng X.G., Tan W.R., Zhou X., Luo S.S., Han X.Y., Zhang D.W., Lin H.H. (2016). Nitric oxide is involved in brassinosteroid-induced alternative respiratory pathway in *Nicotiana benthamiana* seedlings’ response to salt stress. Physiol. Plant.

[45] Xu Z., Jiang Y., Zhou G. (2015). Response and adaptation of photosynthesis, respiration, and antioxidant systems to elevated CO_2_ with environmental stress in plants. Front. Plant Sci..

[46] Rashid F.A.A., Crisp P.A., Zhang Y., Berkowitz O., Pogson B.J., Day D.A., Masle J., Dewar R.C., Whelan J., Atkin O.K. (2020). Molecular and physiological responses during thermal acclimation of leaf photosynthesis and respiration in rice. Plant Cell Environ..

[47] Mujawamariya M., Wittemann M., Manishimwe A., Ntirugulirwa B., Zibera E., Nsabimana D., Wallin G., Uddling J., Dusenge M.E. (2021). Complete or overcompensatory thermal acclimation of leaf dark respiration in African tropical trees. New Phytol..

[48] Scafaro A.P., Fan Y., Posch B.C., Garcia A., Coast O., Atkin O.K. (2021). Responses of leaf respiration to heatwaves. Plant Cell Environ..

[49] Vanlerberghe G.C. (2013). Alternative Oxidase: A Mitochondrial Respiratory Pathway to Maintain Metabolic and Signaling Homeostasis during Abiotic and Biotic Stress in Plants. Int. J. Mol. Sci..

[50] Vanlerberghe G.C., Dahal K., Alber N.A., Chadee A. (2020). Photosynthesis, respiration and growth: A carbon and energy balancing act for alternative oxidase. Mitochondrion.

[51] Manbir, Singh P., Kumar A., Kapuganti J.G. (2022). Alternative oxidase plays a role in minimizing ROS and RNS produced under salinity stress in *Arabidopsis thaliana*. Physiol. Plant.

[52] He Q., Wang X., He L., Yang L., Wang S., Bi Y. (2019). Alternative respiration pathway is involved in the response of highland barley to salt stress. Plant Cell Rep..

[53] Hua D., Ma M., Ge G., Suleman M., Li H. (2020). The role of cyanide-resistant respiration in *Solanum tuberosum* L. against high light stress. Plant Biol..

[54] Watling J.R., Robinson S.A., Seymour R.S. (2006). Contribution of the alternative pathway to respiration during thermogenesis in flowers of the sacred lotus. Plant Physiol..

[55] Wagner A.M., Krab K., Wagner M.J., Moore A.L. (2008). Regulation of thermogenesis in flowering Araceae: The role of the alternative oxidase. Biochim. Biophys. Acta.

[56] Xu F., Yuan S., Zhang D.-W., Lv X., Lin H.-H. (2012). The role of alternative oxidase in tomato fruit ripening and its regulatory interaction with ethylene. J. Exp. Bot..

[57] Liu J., Li Z., Wang Y., Xing D. (2014). Overexpression of ALTERNATIVE OXIDASE1a alleviates mitochondria-dependent programmed cell death induced by aluminium phytotoxicity in *Arabidopsis*. J. Exp. Bot..

[58] Deng X.G., Zhu T., Zhang D.W., Lin H.H. (2015). The alternative respiratory pathway is involved in brassinosteroid-induced environmental stress tolerance in *Nicotiana benthamiana*. J. Exp. Bot..

[59] Avalbaev A., Yuldashev R., Fedorova K., Petrova N., Fedina E., Gilmanova R., Karimova F., Shakirova F. (2021). 24-epibrassinolide-induced growth promotion of wheat seedlings is associated with changes in the proteome and tyrosine phosphoproteome. Plant Biol..

[60] Avalbaev A.M., Yuldashev R.A., Fatkhutdinova R.A., Urusov F.A., Safutdinova Y.V., Shakirova F.M. (2010). The influence of 24-epibrassidinolide on the hormonal status of wheat plants under sodium chloride. Appl. Biochem. Microbiol..

[61] Mishra N., Jiang C., Chen L., Paul A., Chatterjee A., Shen G. (2023). Achieving abiotic stress tolerance in plants through antioxidative defense mechanisms. Front Plant Sci..

[62] Byrt C.S., Munns R., Burton R.A., Gilliham M., Wege S. (2018). Root cell wall solutions for crop plants in saline soils. Plant Sci..

[63] Planas-Riverola A., Gupta A., Betegón-Putze I., Bosch N., Ibañes M., Caño-Delgado A.I. (2019). Brassinosteroid signaling in plant development and adaptation to stress. Development.

[64] Nolan T.M., Vukašinović N., Liu D., Russinova E., Yin Y. (2020). Brassinosteroids: Multidimensional regulators of plant growth, development, and stress responses. Plant Cell.

[65] Basit F., Liu J., An J., Chen M., He C., Zhu X., Li Z., Hu J., Guan Y. (2021). Brassinosteroids as a multidimensional regulator of plant physiological and molecular responses under various environmental stresses. Environ. Sci. Pollut. Res. Int..

[66] Ghosh U.K., Islam M.N., Siddiqui M.N., Cao X., Khan M.A.R. (2022). Proline, a multifaceted signalling molecule in plant responses to abiotic stress: Understanding the physiological mechanisms. Plant Biol..

[67] Kolomeichuk L.V., Khripach V.A., Kuznetsov V.V., Efimova M.V. (2022). Comparison of protective reactions of rape seeds to chloride salination at exposure to epibrassinolide before or during salt stress. Dokl. Biochem. Biophys..

[68] Bhagyawant S.S., Narvekar D.T., Gupta N., Bhadkaria A., Koul K.K., Srivastava N. (2019). Variations in the antioxidant and free radical scavenging under induced heavy metal stress expressed as proline content in chickpea. Physiol. Mol. Biol. Plants.

[69] Singh S.K., Husain T., Suhel M., Prasad S.M., Singh V.P. (2022). Hydrogen sulphide ameliorates hexavalent chromium toxicity in two cereal crops: Role of antioxidant enzymes and proline metabolism. Plant Biol..

[70] Wani A.S., Ahmad A., Hayat S., Tahir I. (2019). Epibrassinolide and proline alleviate the photosynthetic and yield inhibition under salt stress by acting on antioxidant system in mustard. Plant Physiol. Biochem..

[71] Kahlaoui B., Hachicha M., Misle E., Fidalgo F., Teixeira J. (2018). Physiological and biochemical responses to the exogenous application of proline of tomato plants irrigated with saline water. J. Saudi Soc. Agric. Sci..

[72] Merwad A.R.M., Desoky E.S.M., Rady M.M. (2018). Response of water deficit-stressed *Vigna unguiculata* performances to silicon, proline or methionine foliar application. Sci. Hortic..

[73] Guan C., Cui X., Liu H.Y., Li X., Li M.Q., Zhang Y.W. (2020). Proline biosynthesis enzyme genes confer salt tolerance to switchgrass (*Panicum virgatum* L.) in cooperation with polyamines metabolism. Front. Plant Sci..

[74] Özdemir F., Bor M., Demiral T., Türkan İ. (2004). Effects of 24-epibrassinolide on seed germination, seedling growth, lipid peroxidation, proline content and antioxidative system of rice (*Oryza sativa* L.) under salinity stress. Plant Growth Regul..

[75] Moura J.C.M.S., Bonine C.A.V., Viana J.O.F., Dornelas M.C., Mazzafera P. (2010). Abiotic and biotic stresses and changes in the lignin content and composition in plants. J. Integr. Plant Biol..

[76] Ménard D., Blaschek L., Kriechbaum K., Lee C.C., Serk H., Zhu C., Lyubartsev A., Nuoendagula, Bacsik Z., Bergström L. (2022). Plant biomechanics and resilience to environmental changes are controlled by specific lignin chemistries in each vascular cell type and morphotype. Plant Cell.

[77] Choi S.J., Lee Z., Kim S., Jeong E., Shim J.S. (2023). Modulation of lignin biosynthesis for drought tolerance in plants. Front. Plant Sci..

[78] Rakhmankulova Z.F., Fedyaev V.V., Podashevka O.A., Usmanov I.Y. (2003). Alternative respiration pathways and secondary metabolism in plants with different adaptive strategies under mineral deficiency. Russ. J. Plant Physiol..

[79] Wei L.J., Deng X.G., Zhu T., Zheng T., Li P.X., Wu J.Q., Zhang D.W., Lin H.H. (2015). Ethylene is involved in brassinosteroids induced alternative respiratory pathway in cucumber (*Cucumis sativus* L.) seedlings response to abiotic stress. Front. Plant Sci..

[80] Jacoby R.P., Taylor N.L., Millar A.H. (2011). The role of mitochondrial respiration in salinity tolerance. Trends Plant Sci..

[81] Banerjee A., Roychoudhury A. (2018). Interactions of brassinosteroids with major phytohormones: Antagonistic effects. J. Plant Growth Regul..

[82] Saha B., Borovskii G., Panda S.K. (2016). Alternative oxidase and plant stress tolerance. Plant Signal. Behav..

[83] Mizuno N., Sugie A., Kobayashi F., Takumi S. (2008). Mitochondrial alternative pathway is associated with development of freezing tolerance in common wheat. J. Plant Physiol..

[84] Panda S.K., Sahoo L., Katsuhara M., Matsumoto H. (2013). Overexpression of alternative oxidase gene confers aluminum tolerance by altering the respiratory capacity and the response to oxidative stress in tobacco cells. Mol. Biotechnol..

[85] Feng H., Duan J., Li H., Liang H., Li X., Han N. (2008). Alternative respiratory pathway under drought is partially mediated by hydrogen peroxide and contributes to antioxidant protection in wheat leaves. Plant Prod. Sci..

[86] Grabelnych O.I., Borovik O.A., Tauson E.L., Pobezhimova T.P., Katyshev A.I., Pavlovskaya N.S., Koroleva N.A., Lyubushkina I.V., Bashmakov V.Y., Popov V.N. (2014). Mitochondrial energy-dissipating systems (alternative oxidase, uncoupling proteins, and external NADH dehydrogenase) are involved in development of frost-resistance of winter wheat seedlings. Biochemistry.

[87] Garmash E.V., Golovko T.K. (2009). Effect of cadmium on growth and respiration of barley plants grown under two temperature regimes. Russ. J. Plant Physiol..

[88] Wang X., Zhang Z.W., Tu S.H., Feng W.Q., Xu F., Zhu F., Zhang D.W., Du J.B., Yuan S., Lin H.H. (2013). Comparative study of four rice cultivars with different levels of cadmium tolerance. Biologia.

[89] Keunen E., Schellingen K., Van Der Straeten D., Remans T., Colpaert J., Vangronsveld J., Cuypers A. (2015). ALTERNATIVE OXIDASE1a modulates the oxidative challenge during moderate Cd exposure in *Arabidopsis thaliana* leaves. J. Exp. Bot..

[90] Gunn S., Farrar J.F. (1999). Effects of a 40 C increase in temperature on partitioning of leaf area and dry mass, root respiration and carbohydrates. Funct. Ecol..

[91] Rachmilevitch S., Lambers H., Huang B. (2006). Root respiratory characteristics associated with plant adaptation to high soil temperature for geothermal and turf-type *Agrostis* species. J. Exp. Bot..

[92] Shakirova F.M., Bezrukova M.V., Aval’baev A.M., Gimalov F.R. (2002). Stimulation of Wheat Germ Agglutinin Gene Expression in Root Seedlings by 24-Epibrassinolide. Russ. J. Plant Physiol..

[93] Lastochkina O., Garshina D., Ivanov S., Yuldashev R., Khafizova R., Allagulova C., Fedorova K., Avalbaev A., Maslennikova D., Bosacchi M. (2020). Seed Priming with Endophytic *Bacillus subtilis* Modulates Physiological Responses of Two Different *Triticum aestivum* L. Cultivars under Drought Stress. Plants.

[94] Minibayeva F.V., Gordon L.K., Kolesnikov O.P., Chasov A.V. (2001). Role of extracellular peroxidase in the superoxide production by wheat root cells. Protoplasma.

[95] Veselova S.V., Burkhanova G.F., Nuzhnaya T.V., Maksimov I.V. (2016). Roles of ethylene and cytokinins in development of defense responses in *Triticum aestivum* plants infected with *Septoria nodorum*. Russ. J. Plant Physiol..

[96] Beyer Y., Fridovich I. (1987). Assaying for superoxide dismutase activity: Some large consequences of minor changes in conditions. Anal. Biochem..

[97] Yusupova Z.R., Akhmetova I.E., Khairullin R.M., Maksimov I.V. (2005). The effect of chitooligosaccharides on hydrogen peroxidase production and anionic peroxidase activity in wheat coleoptiles. Russ. J. Plant Physiol..

[98] Bradford M.M. (1976). A rapid and sensitive method for the quantitation of microgram quantities of protein utilizing the principle of protein-dye binding. Anal. Biochem..

[99] Tao S., Khanizadeh S., Zhang H., Zhang S. (2009). Anatomy, ultrastructure and lignin distribution of stone cells in two *Pyrus* species. Plant Sci..

[100] Bezrukova M., Kildibekova A., Shakirova F. (2008). WGA reduces the level of oxidative stress in wheat seedlings under salinity. Plant Growth Regul..

[101] Bates L.S., Waldran R.P., Teare I.D. (1973). Rapid determination of free proline for water stress studies. Plant Soil.

[102] Rakhmankulova Z.F., Fedyaev V.V., Rakhmatulina S.R., Ivanov C.P., Gilvanova I.R., Usmanov I.Y. (2010). The effect of wheat seed presowing treatment with salicylic acid on its endogenous content, activities of respiratory pathways, and plant antioxidant status. Russ. J. Plant Physiol..

